# Precision Nanotoxicology in Drug Development: Current Trends and Challenges in Safety and Toxicity Implications of Customized Multifunctional Nanocarriers for Drug-Delivery Applications

**DOI:** 10.3390/pharmaceutics14112463

**Published:** 2022-11-15

**Authors:** Anas Ahmad, Mohammad Imran, Nisha Sharma

**Affiliations:** 1 Julia McFarlane Diabetes Research Centre (JMDRC), Department of Microbiology, Immunology and Infectious Diseases, Snyder Institute for Chronic Diseases, Hotchkiss Brain Institute, Cumming School of Medicine, University of Calgary, Calgary, AB T2N 4N1, Canada; 2 Therapeutics Research Group, Frazer Institute, Faculty of Medicine, University of Queensland, Brisbane 4102, Australia; 3 Division of Nephrology, Department of Internal Medicine, University of Utah, Salt Lake City, UT 84132, USA

**Keywords:** nanotoxicology, biocompatibility, nanomedicine, nanoparticles, drug delivery

## Abstract

The dire need for the assessment of human and environmental endangerments of nanoparticulate material has motivated the formulation of novel scientific tools and techniques to detect, quantify, and characterize these nanomaterials. Several of these paradigms possess enormous possibilities for applications in many of the realms of nanotoxicology. Furthermore, in a large number of cases, the limited capabilities to assess the environmental and human toxicological outcomes of customized and tailored multifunctional nanoparticles used for drug delivery have hindered their full exploitation in preclinical and clinical settings. With the ever-compounded availability of nanoparticulate materials in commercialized settings, an ever-arising popular debate has been egressing on whether the social, human, and environmental costs associated with the risks of nanomaterials outweigh their profits. Here we briefly review the various health, pharmaceutical, and regulatory aspects of nanotoxicology of engineered multifunctional nanoparticles in vitro and in vivo. Several aspects and issues encountered during the safety and toxicity assessments of these drug-delivery nanocarriers have also been summarized. Furthermore, recent trends implicated in the nanotoxicological evaluations of nanoparticulate matter in vitro and in vivo have also been discussed. Due to the absence of robust and rigid regulatory guidelines, researchers currently frequently encounter a larger number of challenges in the toxicology assessment of nanocarriers, which have also been briefly discussed here. Nanotoxicology has an appreciable and significant part in the clinical translational development as well as commercialization potential of nanocarriers; hence these aspects have also been touched upon. Finally, a brief overview has been provided regarding some of the nanocarrier-based medicines that are currently undergoing clinical trials, and some of those which have recently been commercialized and are available for patients. It is expected that this review will instigate an appreciable interest in the research community working in the arena of pharmaceutical drug development and nanoformulation-based drug delivery.

## 1. Introduction

The urgent necessity for the assessment of human as well as environmental hazards of nanoscaled material has motivated the evolution of newer measurement techniques for detecting, quantifying, and characterizing the risks associated with these nanomaterials. Many of these methodologies have enormous capabilities to be applied in diverse arenas of nanotoxicology. In several other events, the restricted dialogues among environmental specialists and human toxicity analysts have confined the fullest utilization of this resource. Vast spectrums of progress have been made in developing the methodologies for nanoparticle analyses in the context of the applications of these methodologies for human or environmental toxicological paradigms [1,2]. Multifunctional nanoparticles possessing distinct features—ascertained by the unique characteristic constitution, particle size, size distribution, surface morphological features, and interfacial identities—have increasingly been integrated into broad areas of pharmaceutical and cosmetics. These characteristics, considerably distinct from the properties of bulk or naïve forms of these materials, regulate the destiny of these multifunctional drug-delivery nanoparticles in the context of the environment along with traits of their interactive behaviors with other biochemical moieties in a living system [3,4,5]. Characterization of nanoparticle samples that possess lower concentrations and various size distributions and are amenable to precipitation, aggregation, dissolution, and the formation of various complexes has become quite challenging for conventional and contemporary analysis tools and techniques. Existing methodologies should be upgraded and newer ones need further development for the generation of authentic and dependable outcomes so as to detect, quantify, and assess the various risks associated with multifunctional customized nanoparticles [6,7].

**Figure 1:**
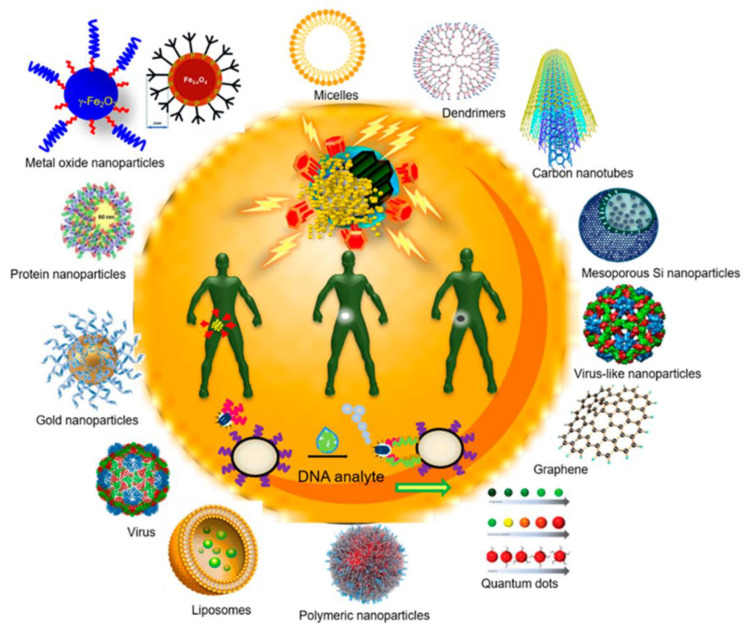
A diagrammatic representation of various types of nanoformulations. The compositions of inorganic materials and polymer-based carriers have been depicted on the outer circumference. Inside, various functionalities which are accomplished by these nanomedicines have been exhibited (reproduced with permission from [17]).

In these recent times, various types of nanoparticle-based platforms have been formulated and exhaustively utilized in a wide spectrum of manufacturing procedures for healthcare-based and other products, viz. drugs, cosmetics, insulations, paints, filters, semiconductor-based devices, cosmetics, and biomedical devices (Figure 1). In the case of nanoparticles, their physicochemical properties, quantum mechanical features, and various biological and biochemical characteristics regulate their interactions with biological organisms. It has also been observed that inhalation, dermatological applications, and oral administration of nanoparticles can introduce various risks to human and environmental aspects [8,9]. Cellular internalization of nanoparticles has been exhibited to occur from the cellular epithelium as well as the endothelium. Hence, the risk evaluations for these nanoparticles have been believed to be an important consideration in the nanotechnology areas. For addressing the enormous and ever-arising numbers and varieties of engineered multifunctional nanoparticles entering these markets, there is a greater need for faster, nonexpensive, and in vivo formalized high throughput screening techniques and approaches derived from in vitro cell-based assays. However, in vitro testing from various laboratories frequently negate each other, and, furthermore, inequality disparity is seen between these in vitro and in vivo consequences [10,11]. Most of these in vitro and in vivo disparities could well be explicated by failures of simpler cell-based systems (which generally employ immortalized or quite abnormal cell lines) for adequate recapitulation of the complex biological milieu from the mammalian organisms. However, there is a high chance of mismatching of the in vitro and in vivo outcomes because of the inadequate and insufficient characterization of engineered nanoparticles and particularly the inability to adequately account for transportation and the fate of these nanoparticles in vitro becomes a major factor in these inequalities [12,13].

Particularly, silver-based nanoparticulate systems exhibit enhanced antimicrobial activities and are among the majorly employed metal-driven nanoformulations that have been detected in various consumer-based goods, viz. cosmetic products, clothes, household items, biomedical tools, and food packages. Because of this preponderance in these consumer goods, silver-based nanoformulations can also be released into environmental surroundings and pose a menace with regard to human health and well-being. In fact, silver nanoparticles are reported to exhibit late toxicities in various beings [14]. Several research reports have been undertaken to address the toxicological issues related to various nanoparticles as well their routes of administration. However, only some definitive tendencies could be established in this regard. This is possibly due to the lack of inclusive or exhaustive assessments of (1) nanoparticle compositions, hydrodynamic diameter, particle size, surface or morphological architectures, and their surface charges in terms of the zeta potential values; (2) densities, heaviness, and stability of their interfacial coatings under the physiological states; (3) an in-depth and complete comparative analysis of (1) and (2) in several of the in vivo model systems; (4) variability with respect to the raw material employed for formulating the nanoparticles and distinctions in their formulation processes; (5) exhaustive immunologic descriptive characterizations [15]. Nanocarriers are administered by intraocular, oral, nasal, or pulmonary and several other suitable routes of administration. In this review, the authors have focused on the toxicity implications of nanoparticles via almost all the routes, which have been common for the wide varieties of engineered and customized multifunctional nanoparticles, and attempted to briefly sum up their interactive capabilities with various bodily components and systems. Some of the key considerations have been included in the context of the preclinical characterizations of nanoparticles formulated for biomedical applications [16].

## 2. Biocompatibility and Safety Issues in Nanomedicine

**Figure 2:**
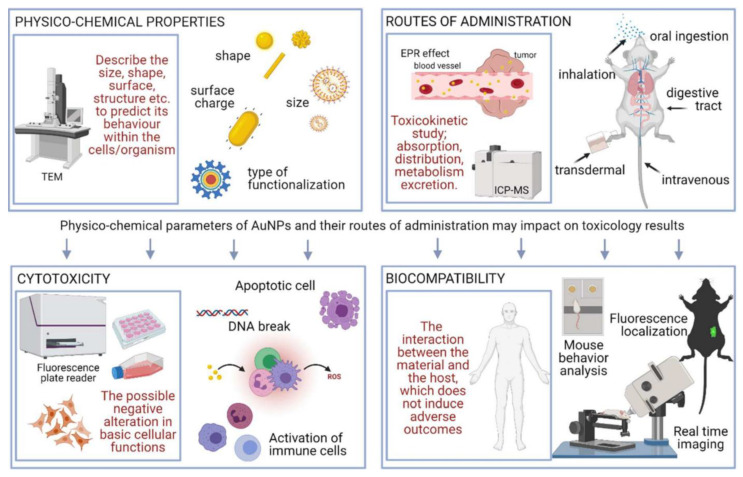
Schematic presentation of the various aspects regulating the biocompatibility and safety implications associated with nanoparticles, e.g., particle size or hydrodynamic diameters, nanoparticle shape and surface morphological characteristics, and routes through which nanoparticles are administered into the patients, considerably regulate the safety and toxicological outcomes including the adverse effects (viz. apoptotic changes, reactive oxygen species yield, abnormality in animals’ behaviors, etc.). These prospects can be assessed on cellular or organismic levels (reproduced with permission from [32]).

Various types of nanoparticles could well be distinguished from each other by their distinct physicochemical characteristic features. One of the obvious methods for the categorization of nanoparticles can also be based on their biocompatibility and toxicity, which can impart a primary component to a nanoparticle’s identity. However, various nanoparticles are composed of many components including the sore and shell made up of different types of materials, the primary contacting portion with the nanoparticle’s external biological environment is often done not by the inner core but by the nanoparticles’ outer coating materials. Therefore, nanoparticles are mostly well-defined in the context of the outer surface of the nanoparticle, which is often composed of a distinct material from that in the core position [18]. The biocompatibility and safety of nanoparticles can well be confirmed in terms of their toxicity inside various cell lines as well as in terms of the histological and serum biochemical considerations (Figure 2). Various studies have demonstrated the biocompatibility of the multifunctional nanoparticles against L929 cell lines, human skin fibroblast cells, and in terms of the histopathological observations of the vital organs such as the liver, heart, lungs, spleen, and kidneys [19,20].

Zhang et al. formulated the nucleic acid functionalized gold nanoparticles (DNA-modified Au nanoparticles for investigating the transportation purpose) and checked the biocompatibility with the help of analyzing the RT-PCR of the messenger RNA transcript levels [21]. Likewise, Hadjidemetriou and coworkers suggest that nanoparticles administered to an organism can be rapidly altered after coming in contact with various biological environments because of the interfacial interactions with several blood components, of which blood or serum proteins have been mostly analyzed. In spite of the extenuation of proteins’ adsorption by the nanoparticles’ surfaces and their functionalization schemes with higher molecular weight deliquescent moieties (including but not limited to glycosylation and PEGylation), presently there have been negligible strategies for the complete elimination of the formation of protein coronas, in vivo nanoparticle biomolecular protein corona formation, and their potential applicability in biological medicines. The potential development of biomolecular protein corona can lead to appreciable improvements in the biocompatibility of nanoparticles and can further ameliorate their toxicity, improve their cell targeting capabilities, can appreciably enhance their drug payload carrying capacities, and can significantly improve their disease detection abilities [22].

Schooneveld and coworkers in their report suggest that hybrid organic and inorganic nanoparticulate carriers composed of polymers and some of the inorganic materials and natural biomolecules can extend a concourse of benefits in nanophotonic paradigms, nanoformulations mediated catalysis, and nanomedicine-related fields. When employed as multifunctional carriers for biomedical approaches, higher biocompatibility has been reported to be achieved, and possibilities of the integration of various (contrast-generating) materials in the same formulation can also further allow their detectability in the multimodal paradigms [23]. Schooneveld et al. in another study state that silica-based formulations are promising carriers for nanoparticle-mediated drug-delivering approaches, gene therapies, and biomolecular imaging techniques. An in-depth realization of pharmacokinetic and pharmacodynamic behavior has become significant for resolving their biocompatibility, biodegradability, and biological applicability-related matters. The have reported a detailed analysis regarding the bare (uncoated or naked) and lipid-coated silica nanoformulations in mice models. Their results were incurred by applying various techniques (including fluorescence-based image analysis, ICP-MS, MRI, CLSM, and TEM) and exhibited that coating of the nanocarriers with lipid-based materials, can enable straightforward functionalization capability and incorporation of several other beneficial characteristic features, and can further also increase the biological applicability and appreciably improve the pharmacokinetic behavior [24].

Therefore, there have been very valid and genuine concerns regarding the nanoparticles’ toxicities, and not much has been known of as how nanosized particles and entities can act in human patients or healthy volunteers. The hydrodynamic diameter or particle size of the nanoparticles, as well as their surface or interfacial characteristics, could provide them access to such localizations which may sometimes not be accessible to large-sized nanocarriers. Surface characteristic features can further impact the biodistribution via various phenomena, viz. non-specific adsorption of various protein molecules from the serum, their removal by the macrophage cells, and bringing about the localized perturbations in biological barriers that can otherwise restrict their accession [25]. A recent example of such phenomena came into the picture where neutral or marginally negative nanoformulations could not hamper the integrity of the blood–brain barrier in the rat model, whereas nanocarriers with high or maximal charges hampered it, disregarding whether these were having either the positive or negative charge [26]. Such reports indicate that further research is needed to fully define the biocompatibility of nanoformulations in human systems. The deliberate establishment of nanoparticles’ toxicity in animals demonstrated no damaging consequences for some (viz., silica-coated magnetic nanocarriers of 50 nm particle size) but toxicological consequences for the other kinds (viz., carbon nanotubes) [27,28]. As expected, hydrodynamic diameter and surface or interfacial characteristic features of nanocarriers further regulate their biological or biocompatibility behaviors and more data requirements further arise for developing and in-depth comprehension of the structure–activity relationship of nanocarriers and their components. Nevertheless, few types of nanoparticles can pass the thorough testing of their toxicological and biocompatibility paradigms so as to obtain regulatory approval. Although each new nanoparticle type is required to be tested for its biocompatibility and toxicity and there are several good reasons for believing that nanoformulations can be finally employed for human use as systemically administered and effective nanomedicines and bio-imaging factors [29,30]. As more and more biocompatibility and biodegradability data, reports, and records are available, further in-depth comprehension of what factors are required for the tuning of nanoparticle sizes and surface or interfacial features for providing biosafety can further help in creating newer and more efficacious medicines for human use [31].

Further, anticancer nanomedicines impart improvised therapeutic characteristics as well as diagnostic platforms to overcome multidrug resistance in tumor cells. Additionally, these anticancer nanomedicines can also surpass the disadvantages of present-day therapeutic modalities which include poor aqueous solubilities of hydrophobic pharmacological compounds, nanoparticle agglomeration, and culminate into improved biocompatibility as well as biodistribution [33,34,35,36]. In the context of magnetic NPs, some of the significant characteristics of magnetic NPs include the induction of magnetic hyperthermia, for drug-mediated, as well drug-mediated bimodal therapy [37] nontoxicity, biocompatibility, injectability, high-level accumulation in the target tumor, and effective absorption of the energy of the AMF [38]. Another strategy for making the NPs more biocompatible for in vivo applications is the employment of eukaryotic L-amino acid-containing peptides due to their inherent biocompatible, biodegradable, and nontoxic characteristics. The biocompatibility of NPs against various cell lines of different origins can be assessed with the help of an MTT assay where the reduction in cell viability upon NPs treatment will be inversely proportional to the biocompatibility of materials from which the NPs are formulated [39,40]. Various biocompatible and safe nanoformulations from various recent studies have been briefed in Table 1. Some biocompatible polymers have also been employed for coating magnetic NPs. Aminocellulose, PLGA, etc. impart biocompatible characteristics to NPs and make them less toxic [19]. Further, grafting the pharmacokinetic modifier, viz. polyethylene glycol, onto nanomaterials’ surfaces via the self-assembly process can appreciably improve the biocompatibility of NPs and prolong the blood residence timings, which may be because of the reduced protein adsorption or circumventing the cellular or RES uptake in the reticuloendothelial system [41]. The magnetic nanosystems which exhibit excellent biocompatibility and biodegradability are counted under smart drug-delivery systems (SDDSs) [42,43,44]. Sometimes, a hemolysis assay is also performed to assess the biocompatibility of nanomaterials and the absence of hemolysis by NP even at higher doses represents the excellent biocompatibility of NPs. In the context of establishing the biocompatibility of magnetic NPs, if the body weight of the treated animals shows normalized variations and there is no significant tissue destruction or other side effects in cardiac, hepatic, splenic, pulmonary, renal tissues, then it is considered to confirm the established biocompatibility of nanomaterials and exhibit the good tolerance towards NPs [45,46]. Among various NPs, mesoporous silica nanoparticles (MSNs) have also been widely researched as smart drug-delivery systems because of their specific mesoporous characteristic, the appreciable potential for their surface modifications, and better biocompatibility profiles [47].

Likewise, nanoemulsions have also been used as excellent drug-delivery nanovehicles and other pharmaceutical applications due to their optimum size, shape, and biocompatibility profiles [48,49]. One of the common preclinical safety and toxicology assessments of nanoemulsions analyses their interaction with RBCs and platelets—which are the chief cells involved in the process of blood coagulation. The surface modification of nanoemulsions with various functional groups can improve their blood compatibility profiles [14,50,51]. For nanoemulsions, their safety and toxicity can be evaluated in vitro following the various available standardizations, viz. ASTM-E2526-08 as well as ISO-10993-22; however, it has also been suggested to apply relevant target cell lines, including the whole blood as well as peripheral-blood mononuclear cell lines and also specified immunological cellular subtypes, e.g., T cells, Raji-B lymphocytes, and THP-1-human monocyte cells for more clearly defining the exposure as well as dose–response relationship [52,53,54]. The bioavailability, degradability, and cellular as well organismal biocompatibilities are substantially considerable characteristics of nanoemulsions which have drawn the attention and care of researchers to apply these as nanotechnology-based pharmaceutical drug-delivery agents in the treatment of various disorders [55,56,57].

## 3. Current Trends in Safety and Toxicity Assessment of Nanoparticles In Vitro

The exponential increase in the usage and production of nanoparticles (NP) raised perilous effects on human health as well as the environment. They are highly vulnerable to biomedical applications and several studies are going on for nanotoxicity assessment [73]. Among all types of techniques, in vitro assessments are considered to be the most reliable, thrifty, highly accessible, and free from animal usage [74]. During the assessment, different cell lines are utilized such as hematologic, tumorous, hepatic, and neuronal. In vitro studies involve cell viability assays; cytotoxicity assays for identifying oxidative stress; cell stress assays for gene expression [75]. Inclusive detection by the application of in vitro tools and methodologies is one of the most broadly consented methods and numerous attempts utilized for cytotoxic research and explorations. These techniques differ in terms of their panache of detecting the cell death paradigms. Some of the most popular assays for in vitro toxicity assessment are MTT, the trypan blue dye assay, the Comet assay, and the 2’, 7’-dichlorofluorescein diacetate assay [74].

As per previously published articles, it was summarized that elevated reactive oxygen and nitrogen species production, inflammation, and cytokine overproduction results in apoptosis, autophagy, and necrosis cell death mechanisms [75]. These remain the major markers observed as nanomaterial-induced toxicity [76,77,78]. In past years, remarkable advancements have been made in the field of molecular biology to offer various modern techniques to evaluate the toxic effects of nanoparticles.

The factual advancement of research and explorations can be demonstrated by its clinical translation capability and this backdrop could well be proved in recent times by the handiness and accessibility of almost 50 types of nanoparticulate formulations in the cosmopolitan markets which flourished in markets, raising around USD 140 billion from 2015–2016 from almost USD 50 billion from 2008–2009, where anticancer medicinal approaches have contributed the major portion. Somewhere around 2019, human serum albumin nanoparticles encapsulating the paclitaxel with the brand name Abraxane reached an estimation of around USD 970 million in overall revenue [79,80]. Once the initial approving of Doxil (liposomal formulations containing the drug doxorubicin with a particle size of approximately 100 nm) was granted by the United States Food and Drug administration in 1995, explorations on nanomedicinal approaches expanded exponentially throughout the scientific community and a present look on clinical trials website results in almost 500 reports on several indicants which include cancer biology, autoimmunity, infectious disorders, cardiovascular studies, hormonal imbalances, and orthopedic disabilities in various phases of clinical studies, establishing these as making an improved and better future world. However, a constant necessity has always arisen to minimize the gaps in the translation of these nanomedicines from the bench side to upscale and industrialized productions and to achieve clinical applicability [80,81].

**Figure 3:**
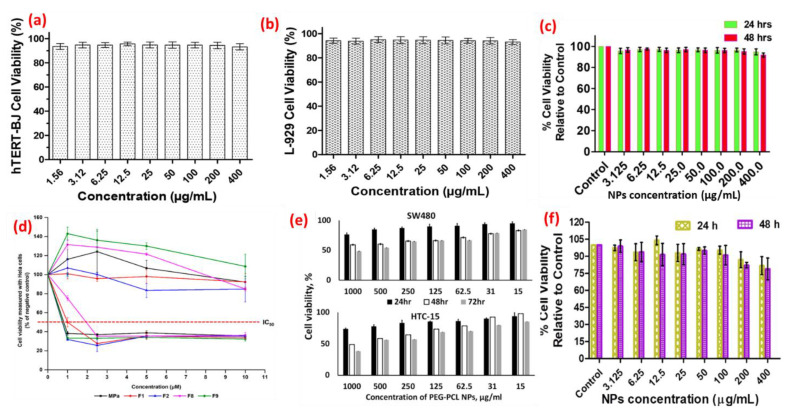
Safety and toxicity assessment of different nanocarriers in vitro. (**a**–**c**) Assessment of safety and toxicity of blank and drug-loaded chitosan NPs in dose-dependent and time-dependent manner (reproduced from [82] with permission from the American Chemical Society). (**d**) Viability of two cancer cell lines (HeLa and A549) treated with free compound and its loaded nanocarriers, F1, F2, F8, and F9 (reproduced from [84]). (**e**) Cytotoxicity analysis of PEG-PCL NPs with encapsulated Man-PEI/plasmid complexes in SW480 and HCT-15 cells, reproduced from [83]. (**f**) MTT assay for cytocompatibility of PCL-AC-gel NPs towards normal human foreskin fibroblasts (hTERT-BJ) cells (reproduced from [19] with permission from the Royal Society of Chemistry).

The nanoparticles may induce dose-dependent as well as time-dependent cytotoxicity in various cell lines in which their in vitro safety and toxicity are being assessed. This cytotoxicity is assessed in terms of the decrease in cell viability by employing various cytotoxicity assays, the chief among those being the MTT cell viability assay. Figure 3 gives an idea of where different NPs have been used for assessment of their safety and toxicity profiles against various cell lines both in time-dependent and dose-dependent manners and percentage cell viability (either absolute or relative to some control), establishing up to what dose the nanoparticles are safe and at what doses their toxicity starts appearing against the tested cell, which is indicated by the corresponding reduction in the cell viability [19,82,83,84].

Recent epidemiologic explorations on nanomedicinal formulations and their carcinogenicities have so far been indeterminate. Databases required for assessing the carcinogenic potential of nanoparticles have similarly been quite deficient. Evaluations of carcinogenicities and their significance with respect to humans have often been unsettled in terms of quantitative and qualitative outcomes. In qualitative contexts, smaller sizes, absorptive capacities, capabilities, times, and durations of retention; their biodistribution overcoming most of the biological hindrances; and subcellular or molecular-level interactions have all been found of having major influences [85]. Compared to their bulky materials, the carcinogenic potencies of nanoparticles are higher due to their tiny particle sizes and correspondingly higher surface areas, i.e., carcinogenicities of nanoparticles and their bulk counterparts are fundamentally quite distinct. As the orbicular output or yield of nanoparticle production has been advancing day by day, newer nanoparticles possessing improved and amended characteristics are anticipated soon. Some of the major risk assessment approaches and their advantages as compared to conventional methods have been mentioned in Table 2.

In the context of various nanoformulations, various characteristic features such as structures, sizes (distributions), shapes, and specific surface characteristics (viz. coatings) have been preferred for pharmaceutical applications and are non-toxic. Regarding the structures, nanoparticles either made up of organic polymers or metallic inorganic nanoparticles coated with organic, natural, or nature-mimicking polymers have often been reported to be non-toxic and highly biocompatible for pharmaceutical applications [99,100,101,102,103].

It has generally been observed that nanoparticles lesser than 300 nm have been found to be the least toxic and are biocompatible with most of the cell lines as well as blood cells. Furthermore, the toxicity of nanoparticles is dependent upon their size but majorly depends upon the dose in which nanoparticles have been administered and cells or tissues have been exposed to the enhanced concentrations of nanoparticles [8,104,105]. Gold nanoparticles of 13–25 nm size, studied by Fede et al. in 2015 [106] are reported to be non-toxic in various cell lines. Fan and coworkers have studied gold nanoparticles and gold nanorods for cancer cell cytotoxicity and found them nontoxic against normal cells in optimized concentrations [107]. Auffan et al. in their report have described that the pharmacological applications of nanoparticle size as high as 100 nm have been found to be non-toxic and biocompatible against various cell lines in optimized doses [108]. Malysheva and coworkers also study the cytotoxicity of nanoparticles with various sizes (as high as 100 nm) in different concentrations, and toxicity was majorly dependent upon the charge of the metal ions rather than the size of the nanoparticles [109].

With respect to the shape of nanoparticles, various shapes have been formulated and applied for pharmaceutical applications and nanoparticle shapes include nanospheres, spherical polymeric micelles, hollow polymeric nanovesicles, nanoworms, nanorods, and nanostars. Hinde and coworkers have demonstrated that it is actually the shape-derived aspect ratio of nanoparticles, rather than the nanoparticles which decides the toxicological features of nanoparticles. The nanoparticle shapes possessing higher aspect ratios exhibit higher toxicities in comparison to the nanoparticle shapes with lower aspect ratios [110].

In the context of the surface coating of nanoparticles, surface-coating procedures using polymers, lipid bilayers, and environmentally responsive molecular coats have been formulated for the loading, retaining, protecting, and releasing loaded therapeutic drug cargo, and imparting stability, hemocompatibility, and biocompatibility in physiological media and blood microenvironment [8,111,112]. Silsesquioxanes and polyethylene glycol (PEG) coated nanoparticles [113], poly(N-isopropylacrylamide-co-methacrylic acid) [114], chitosan [82], eudragit polymers [115], gelatin or casein polymers [116], lipid coatings, polyethylene imine coating, cetyltrimethylammonium bromide (CTAB), polyacrylic acid (PAA) [111], etc., have been widely employed for regulating and minimizing the toxicity of nanomaterials and ensuring and maximizing their biocompatibility against various cell lines and tissues.

## 4. Risk Assessment of Nanocarriers In Vivo

The nature and interactions of nanocarriers help in understanding the assimilation pathways followed to enter into the organisms. As it became essential to assess the potential hazard of nanocarriers, the risk assessment of the nanocarrier’s chemical moiety has been the relevant approach to determine the possible risks. Nanocarrier risk assessment is an important process where scientific principles are applied to designate hazards linked with environmental and human exposure to chemicals [74]. To assess the risk of nanocarriers, scientists have performed several in vivo studies in rodent animals to evaluate acute and chronic toxicity. In vivo methods hold their primary standards for toxicity assessment. In this, nanocarriers are introduced into the animal body and monitored for pharmacodynamics. These types of studies crucially needed signs of progress. These studies entail real-time analysis where results remain coherent with human body functioning [117].

Systemically administered nanoparticles have the capability to cause the infusion reaction in clinical subjects, an untoward reaction that generally either prolongs or stops the clinical translation potentials of these nanomedicines. This reaction can exhibit itself in terms of rash, chest pain, chills, fever, rigors, etc., or sometimes trouble with respiratory ventilation, while in some rare cases, these could also prove to be lethal. Identification of the risks of infusive reaction in the earlier stages of the drug developmental phases can aid in the mitigation of the vital safety detrainment when nanoformulations are translated into clinical stages, and hence can save both money and time of developers, and protecting the patients from significantly harmful consequences [118,119]. Hence, approaches for evaluating the toxicities have become quite essential with respect to human hazards as well as risk assessments and these toxicological outcomes can well be researched in silico, in vitro, or in vivo. Production of reactive oxygen species is often the causative phenomenon of probable toxicities of customized multifunctional nanoparticles, leading to both immunological toxicities as well as genetic toxicological outcomes. However, newer toxicological hallmarks, specifically those of epigenetic anomalies, are also considered in investigations. This implies that several toxicological assessment tools and techniques could be chosen. Nanoparticle toxicological risk evaluations also consider several exposure paradigms (viz. ingestion, inhalational, transdermal absorptive potential, or sometimes via the injection routes) and hence can vary appreciably depending upon the source as well as the routes of nanoparticle exposure. For example, the progression in the nanoparticle-mediated drug-delivery approaches required to undergo safety testing which is quite distinct from those required for food-based products or additive substances [120,121].

In comparison to in vitro assessment, in vivo results remain more relatable to human beings. It could be explained with the help of various crucial factors such as hormonal alterations and cell–matrix interaction. Additionally, chronic and long-term toxicity effects cannot be measured in in vitro studies [80]. During in vivo experiments, dosing is defined by the nanocarrier’s exposure to the body which remains a technical challenge due to its minimal size. Moreover, nanocarriers are highly vulnerable to agglomeration due to larger surface area, which ultimately leads to unwanted results. Nanocarriers interact with cell proteins which alter their properties, interface, and biodistribution. Further, the protein endures conformational changes which result in transformed signaling pathways. Therefore, one must consider the different interferences of nanocarriers prior to analyzing the toxicity [122,123]. A group of scientists utilized gold nanocarriers and analyzed their effect with scanning electron microscopy (SEM), cytokine assays, and RT-PCR. They reported the compatibility of gold nanocarriers, which was evidenced by insignificant alteration in cytokine expressions [122]. Rizzo and group performed in vitro and in vivo correlation studies using zebrafish embryos. They assessed the uncoated nanomaterial with biocompatible coated polymers such as pure ultra-small superparamagnetic iron oxide (USPIO) as well as those coated with the flavin mono-nucleotide USPIO (FLUSPIO). The results described the higher toxicity of USPIO as compared to the remaining two NCs. In other studies, silver nanocarriers have shown stage-dependent toxicity against zebrafish embryo tissues. Every embryonal developmental stage such as cleavage, gastrula, segmentation, and hatching stages showed varied nanocarrier concentration ranges from 3.5 pM to 8 pM [124,125]. Unremarkably, the cleavage and gastrula stages revealed the most deformed embryonic stages which were evidenced by altered gene transcription and signaling pathways.

**Figure 4:**
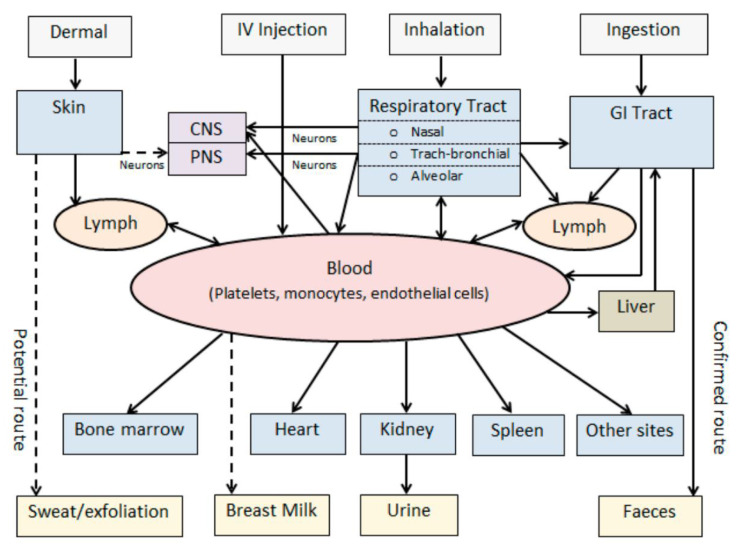
Schematic representation of the various routes of nanoparticle exposure and their biokinetic pathways helpful in nanoparticle safety and toxicological implications in vivo; reproduced with permission from [127].

Hence, these studies help in understanding the pharmacokinetics and pharmacodynamics of nanocarriers. The research articles presented in vivo testing showed variable data depending upon the animal species, analyzed selected end points, and study duration. Furthermore, studies reported important endpoints such as clinical signs of infection, altered serum albumin/creatinine ratios, variation in organ weights, and histopathological lesions in various organs [126]. In recent years, the scenario attracted in vitro and in silico techniques in order to lessen animal usage. Several approaches for the assessment of the safety and toxicity implications of nanoparticles have been depicted in Figure 4.

## 5. Challenges in the Risk Assessment of Nanocarriers

Today, applied science has flourished. However, concerns about potential health effects have arisen for untested materials [128]. In fact, it became essential to outweigh potential human health over commercial and societal benefits [129]. Thus, several international efforts have been planned and/or executed, especially in the European Union and the United States in order to understand possible hazards related to consumer vulnerability [128,129,130]. One of the major hazardous effects of nanoparticles includes pulmonary toxicity due to the ultrafine particles. These particles remain more dangerous as compared to larger ones. However, real advancements have been made so far in order to implement environmental and health-based protocols to address nanosafety issues [128]. To conduct toxicity studies, the characterization of chemical substances exerts a crucial role [131]. To date, various techniques are being used to characterize the different forms of nanocarriers such as solution, powder, and film. Still, the risk assessment of nanocarriers remains an un-met challenging task for the scientific community. The physical properties such as varied size and shape, crystallinity, agglomeration, solubility, and porosity of nanoparticles make it extensive to test as compared to any other formulations [74]. “Size” remains one of the important factors which change the action of nanocarrier together with variegate activity within a living system. The dynamic light scattering (DLS) technique, transmission electron microscopic (TEM) analysis, and assessment by the Brunauer–Emmett–Teller (BET) are various techniques utilized to determine size distribution. Even so, it is difficult to find appropriate information on size distribution due to different principles followed by different techniques [74,130]. Apart from it, sample handling and preparations remain the additional factors responsible for measurement differences. Ultimately, it brings about misperception in finding the absolute nanocarrier size; hence, the researcher must acquaint with the technicality of measurement methods [132].

Because of the chemical or structural ramifications of customized multifunctional drug-delivery nanoparticles, conventional regulative methods could sometimes not be suitable to assess and evaluate their toxicological as well as safety implications. Furthermore, the necessity for placebo-controlled clinical trials as well as therapeutic regimens also requires further reconsideration. The US-FDA also inducted the Nanotechnology Regulatory Science Research Plan to address some of the bigger scientific lacunae in terms of the comprehension, methodologies, and techniques needed for making the regulatory evaluations of nanomaterial-based particles and various other nanoformulations [133]. This initial step has led to the defining of five significant criteria needing to be addressed: physiochemical evaluation and assessment, preclinical model developments and establishments, risk as well as biosafety assessment, risk-based nanomaterial characterizations, and communicating the risks associated with the nanomaterials. A straight illustration of the outcomes of this initiation is establishing the Nanotechnology Characterization Laboratory, which can perform a complete assessment and characterization of the nanoformulations obtained from academic settings, government functionaries, and industries. A standard evaluation cascading can be undertaken for testing the physiochemical characteristics, preclinical toxicological implications, and pharmacology, along with their effectiveness both in vitro and in various animal models [134,135].

An inclusive characterization and assessment of nanoparticles and their intractability with cellular and tissue components are necessary and significant for their applicability in drug-delivering paradigms. While efficacy and safety assessments have become vital for the clinical translation and development of nanoparticles, in-depth comprehension of the nanoparticles’ mechanism of action can lead to the opening of newer disciplines in drug carriers’ functionalization and customization. Identifying the vital dimensions which can prove enough for achieving the prolonged targeting become critical for the mitigation of the risk associated with the higher complexities of these nanosystems [136]. However, the virus-like hydrodynamic diameter or the particle size of these nanoparticles and their enhanced complexities in comparison with the synthetic delivering schemes (e.g., liposomal nanoformulations), which can are partly conducive to better drug-delivering capacities of customized and multifunctional drug-delivery nanoparticles, make a complete evaluation and quality assurances an ever-challenging task [137]. The purity and identification offer various bigger disputes in the analyzing tools and techniques, and sometimes because of the unfitness of the characterization paradigms, the complete settings culminate into various significant risk factors; these circumstances are required to be comprehended in contexts where nanoparticles generally constitute a cell-free therapeutic paradigm. Standardized characterization tools (e.g., nanoparticle tagging analysis, cell-imaging flow cytometry, and detecting the various component systems by biochemical measures (viz. bio-imaging, Western blotting, and flow cytometry)), implicate the particle size measurement of these nanoparticles [138,139].

Further, scientists must ensure the reproducibility of nanocarriers which helps in relying on ultimate results. Moreover, skilled professionals and highly sophisticated instruments are required to characterize the nanocarriers, and the physicochemical properties of nanocarriers are imperfectly understood [140]. On account of sophisticated lab facilities, limited resource availability binds the researchers to work with available resources [141]. Restrained research methodologies such as time-duration and dose-response issues could not be resolved in inadequate facilities. However, the major analytical challenges involve nanotoxicological pathways altered from the correlated to the abortifacient views, to overcome nanocarrier encumbrances for clear in vitro and in vivo assessment.

## 6. Regulatory Guidelines and Legal Aspects of Nanotoxicology

There have been very many claims that drug-delivery nanoparticles can cause harm to the environment and to humans as well; therefore, more studies and explorations are required to be undertaken for a clearer comprehension of the untoward effects of these nanoparticles. Regulatory authorities of various regions and countries along with international assemblies need to elevate their regulatory guidelines in the context of safety, toxicity, and good manufacturing as well as laboratory procedures. Large amounts of investment funds previously caused the advancements of various research institutions, professional and regulatory agencies, pharmaceutical firms, and testing labs for the creation of standardized operation protocols for assessments and evaluations [142]. The straightaway aims of nanoparticle regulatory risks evaluation as well as in their management are ensuring their safety in the context of their proposed applicability. Particularly, accurate definition, terminologies and classifications, and their labeling are required for avoiding the local, regional, or sectoral distinctions in terms of how the nanoparticles are assigned specific definitions, and also the categorization of their subcategories, to which particular care and regulating evaluations are compelling [143].

Regulating and standardization authorities (e.g., the US-FA, EPA, European Chemicals Agencies, OECD, and ISO) have been seriously committed to developing and validating the methodologies to characterize various intrinsically defined characteristics and media-dependent external features of nanoparticles and to describe their routes of exposures and various related hazards exhibited by nanoparticles to environments as well as human health. Questions including “which testing parameters are dependable for the identification of the possible health implications of nanoparticles” and “how the knowledge and comprehension can be translated in the context of regulatory requirements” need to be elucidated to avoid false positives and negatives and misrepresentations of safety or toxicology data for the nanoparticle safety research [144].

Although there have been unfathomed stakes among the policy-making agencies and scientific communities for moving from animal-oriented individualized toxicity evaluations towards structured hazard assessment and screening schemes, the deficiency of pragmatic steering regarding the accorded applications of non-animal assessment methodologies in the regulatory settings has culminated in lower regulatory and industrial adoption [145]. The generation of dependable nanoparticle toxicity data is often a chief issue, but the translation of these pre-perspective explorations culminating into regulating consequences is a completely distinct job. Generally, regulators’ initial interests regarding the want of nanotoxicology data were replaced by the deficiency of regulations pertinent data. Although larger quantities of nanotoxicology data were generated in previous decades, most of this data suffers from a lack of consistency and various other concerns between their duplicate samplings, methodologies, analyses, or labs [146,147].

For regulation proposals, biological nanoparticles have been under the models set by EMA. These frameworks are the regulatory plans and schemes for following-on the nanoparticles, which also undertake testimonials for the relative qualities, preclinical and clinical cases, and reports [148]. The industries and agencies often require scientific proposals and then these cases are analyzed by the European Medicines Agency. Many times, nanomedicine frameworks become the basis for regulatory aspects of agencies, since they possess few common characteristics: viz., structures cannot be completely evaluated and in vivo activities rely upon fabrication processes or accordingly, comparability requirements for establishing during their life cycle, as occurring in case of nanoparticles. Furthermore, in the case of other multifunctional nanocarrier groups including liposomal formulations, glatiramoids, and Fe-carbohydrate-based nanocomplexes, there have been drafted several regulatory schemes and plans that may aid regulatory agencies and authorities in the creation of the final models for various nanoparticle groups and categories [149,150].

Freshly gained data and information could be employed for regulatory projects when fundamental policy formulators as well as lawmakers can fully interpret, translate, and can draw inferences from it. Therefore, the central feature for the successful consolidation of newer data and information in the context of regulatory frames and models and deciding procedures is to clearly exhibit the dependability and significance of their effects and consequences for regulation objectives [151,152]. For the facilitation of information flowing from formulation to policy applications, the following roadblocks and limitations are required to be taken into account:

1. Allowing for comfortable and easy-going accession and admittance to the information and data;
2. Generation of confirmable, coherent, logical, and high-quality data;
3. Fosterage of cross-disciplinary and co-operative research and explorations;
4. Development of the working and practical relations among decision-making authorities, regulatory agencies, as well as several other significant partners;
5. Increase the openness and receptiveness of regulatory agencies and authorities for the newer data, information, tools, and techniques [153].

## 7. Translational Development and Commercialization Potential

Although customized drug-delivery multifunctional nanoparticles have exhibited appreciable remedial benefits for a wide spectrum of pharmacological and theragnostic applicability, their clinical translational paradigms have not advanced as speedily as the profuseness of encouraging preclinical outcomes could have hinted. For moving any of the functional nanoparticles from the bench side to the bed, various respective experimental and observational demanding situations are required to be answered [154]. From the biomedical viewpoint, these challenges implicate considerations and examinations focusing upon the comprehension of their in vivo fate and other biological interactions of nanoparticles with blood components such as serum proteins and blood cells, various body tissues, cellular and subcellular components, and intracellular compartmentalizations in the host’s bodies both in health as well as in diseased conditions [155]. For these drug-delivery nanoparticles to be translated into the clinic, or to possess clinical translational capabilities, the complexities in their formulations and developmental paradigms also must be minimized as much as possible to create a system that is capable of achieving higher reproducibility in terms of nanoparticle synthesis and characterizations [81].

Furthermore, nanomedicine’s clinical translation has always been a costly and time-taking procedure. Nanoparticle technologies often demonstrate quite larger complexities as compared to conventional or traditional formulation strategies which implicate the dispersion of free drugs in bases (viz. tablet, capsule, or injection formulations). Various important issues in the context of the clinical translational developments of nanoparticles have been mentioned in Table 3, and we take into consideration various biological issues, scaleup and manufacturing challenges, biosafety, and compatibility issues, intellectual property rights, governmental regularizations, including their cost effectivity as compared to traditional or conventional therapeutic paradigms [156]. These elements could well enforce considerable hurdles and problems restricting the appearance of nanoparticles on market shelves, irrespective of the fact that these are pharmacologically effective. Conventionally, drug-delivery nanoparticles’ developmental processes have been relying upon formulation-derived approaches, where newer drug-delivering platforms are initially customized and tailored and are then characterized in terms of their physicochemical characteristic features [157]. Only when someone attempts to align and adjust nanoparticles along with some of the pathophysiological usage, it is often observed that limits and restrictions in their clinic-based translation of these nanosystems could well easily be discovered and identified [158]. The comprehension of relationships between nanomedicine and technologies also includes the understanding of the various influences of the pathology of the diseases on nanoparticles’ bioaccumulation, biodistribution, systemic retentive capacities, and pharmacological efficacies, including biopharmaceutical correlative paradigms among various drug-delivery systems and their characteristics, and in vivo behaviors in various animal models as compared to the human systems are significant determiners of translational success of customized and tailored multifunctional drug-delivery nanoparticles. Therefore, the application of some disease-derived plans by formulation and development of these nanoparticles which can be capable of exploitation of the pathological perturbations in disease states can be proposed for improvements in terms of the clinical translation of these nanoparticles [159,160].

Recently, some scientific advancement achieved as an answer to COVID-19 disease has efficiently streamlined the several years and decades of the challenging situations and obstacles in clinical translation pathways confronted by nanoparticle-based medicines. Indeed, rapidly growing and quick approvals, which uphold the highest measures for safety and toxicity assessments and also need lesser bureaucratic hurdles, have yielded appreciable hopes in terms of the future of nanoparticle-based medicines (Đorđević, S. et al., 2021). Back in 2005, pharmaceutical technologies and industries functioned along with the regulatory agencies for setting up the European Technology Platform on Nanomedicine (ETPN), which was the first step in addressing the applicability of nanotechnology-based formulations in health and disease. The ETPN aimed at the creation of circumstances for flourishing and productive translations of nanomedicine-based products by forming and affirming the public funds and finances in the bright areas of nanoparticle-based formulation, research and development, and planning and formulating the unique proficient infrastructures called the Nanomedicine Translational Hubs. ETPN Nano Medicinal Translation hubs offer customized instructing by the Translation Advisory Board, nanomedicine characterizations by NCL, as well as GMP manufacturing via pilot-based original platforms [161,162].

For multifunctional drug-delivery nanoparticles which integrate presently existing traditional or conventional medicines with newer carrier-based technologies, or which integrate existing medicines with conventional or traditional carrier-based technologies for some newer biomedical, diagnostic, or therapeutic applications, intellectual property situations have become further perplexing with more complicated drug delivering platforms, e.g., those that can integrate commercial targeting moieties (viz. antibodies) or coating materials (viz. Eudragit) which were possessed by various other firms and industries. Intellectual property strategy can further involve several patents of any technological platform and require cross-licensing placements [163,164]. Therefore, newer intellectual property exercises and processes are needed for the simplification of the pathways which include the stages starting from inventions to the commercializing steps for reducing the time as well as expenses needed for the negotiations of collaborative efforts and license or permit arrangements. The complications with nanoparticle technology have culminated into what is known as the “patent thickets”, which could well result in costlier litigation and can hamper or completely stop commercialization attempts [165]. Therefore, considerable improvements in the clarity on intellectual property as well as patents circumventing the nanoparticles technologies in health or diseased nanomedicines is needed and would require the involvement for implementing the cosmopolitan regulative rules and laws as well as policies which can well be tailored and customized for this niche commercializing arena [166].

From the commercialization viewpoint, the essential infrastructure, in-depth comprehension of nanoparticles, and a set of capabilities needed for the business development of nanoparticles have currently not been constituted in most of the pharmaceutical industries. Such elements are also needed to be taken into consideration while performing the assessments and final cost-effectiveness of nanomaterials as compared to present conventional or traditional therapeutic approaches [167]. Additionally, when one wants to render nanoformulation and development commercially viable and feasible, they should be counterbalanced by therapeutic or pharmacological values of the appreciably improved in vivo activities. Further, while the academicians’ communities, with a good reason, may feel shy from commercialized viabilities in pursuit of their research-based or explorative destinations, it has become quite evident that ordinary drug developmental clinical programs can cost as high as USD 400 million (and generally surpassing USD 1 billion, which includes the marketing costs as well), so investing by the venture capitalist firms or companies or larger pharma industries becomes quite inevitably clear [168,169].

For improving the opportunities for academic nanomedicine-based designs to be translated into the clinics and to be carried forward towards the formulation and product developmental stages, it requires the advocacy for some more active attitudes for the isolation and endorsements of those platforms and settings which actually possess quite viable and higher commercial as well as clinical potentials. Further, there are key development challenges initiated from wider commercialization feasibilities, and taking into account several other core clinical consequences and ultimately directing the preclinical as well as key pharmaceutical prospects needs to be considered prior to engaging in clinical translational objectives [170]. These clinical translational and/or commercialization challenges could well be exploited as grading boards to evaluate the clinical translational capabilities, and the means for developing sufficient risk-mitigating schemes, which investors, capitalists and financers, and commercialization companies generally would like to observe at comparatively earlier development phases [171].

The initial and foremost challenge for the consideration prior to any of the nanoparticle-based medicines being undertaken for the developmental process can correlate with the commercialization as well as practical feasibilities from the viewpoint of their elementary or chief targeted indications. In this regard, both the capability of improved patients’ welfare and the sample size of the ultimate patient populations are significant. Considerably amended patients’ benefits could also include increased pharmacological effectiveness, lesser toxicological implications, or merely by nanoparticle-based medicinal formulations necessitating lesser frequency doses or enabling a better commodious administrative route than comparative drug products (and hence encouraging the patients’ compliance) [172]. In these cases, the more absolved the benefits are, the easier the considerable rate premiums could be vindicated after the entry of nanoparticle-based drug products into the market. This, while combined with patients who can ultimately consume these, can determine probable markets as well as the products’ sales, and will then be able to entice financers and other commercial companies to invest [173,174].

Further, a number of approaches can be undertaken to maximize the safety and minimize the toxicological impacts of nanoparticles on health. This can start with the “safety by design” strategy which is considered one of the initial points when novel nanoparticles are engineered, and rigorously adopting the principles and practices of drug discovery and development throughout the entire processes of products’ formulation and development (F&D) [175,176,177]. The rationale behind the “Safety-by-Design” (SbD) approach is the minimization of the unwanted and adverse effects of nanoparticles through the implementation of knowledge of nanoparticles’ undesired and harmful effects on human health in the approaches for the designing of desired nanoparticles. The realization of the optimized applications and employability of NPs in biological systems and for moving forward with their translational applications in clinical settings would necessitate the rationalized designs which are determined by how the physicochemical characteristics of nanoparticles can affect their destiny and pharmacological effects in the living systems [178]. Additionally, the probability of the introduction of multiple modalities in the same NPs with minimized interferences can render these NPs more attractive and beneficial. Such NPs can efficiently function as the multimodal contrast media for imaging as well as for therapeutics to provide complemental information in the disease treatment as well as diagnosis. Another approach is the site-specific delivery of nanoparticles and in this context, it has recently been demonstrated that systemic delivery of RGD, which was conjugated with the PEGylated gold nanoparticles (RGD:AuNP) in combinatorial approach with the image-guided radiation therapeutics causes the specified targeting of cancerous blood vessels. This site-specific destruction of tumor endothelia could improve the radiation therapy results and that too with minimal off-target toxicological implications [179].

Furthermore, there is the combination of nanotherapeutics with photothermal therapy (PTT) a light-dependent therapy formulated for the eradication of cancers through the converting of light energy into heat for the optical absorption phenomena. In this context, the photothermal absorbers should possess the optimized absorbances within initial 650–850 nm or subsequent 950–1350 nm biological windows, when the light will interpenetrate intensely inside the cancerous tissues and will cause minimal toxicity to the adjacent healthy tissues [180]. Computational nanotoxicological studies and algorithm-grounded strategies for the prediction of toxicity, safety, and the pharmacological efficacy of NPs have also been emerging. The ongoing development, as well as the establishment of computational techniques and precise and accurate in silico forecasting in the context of the safety and biological destiny of NPs designs, could well be accomplished. Integration of computational tools as well as modeling techniques at the NP-design levels can be appreciably beneficial in the NP advancement and their translational successes in the clinic. Additionally, by improving and enhancing the encapsulation efficiencies of the drugs and nanoparticles and improving the drug loading capacities of these nanocarriers, the employment of vehicle materials can be considerably minimized and, consequently, the dose of the drug can remain the same and can further be reduced, and, therefore, the nanotoxicology caused by vehicle materials would be considerably minimized. Additionally, core-shell structured nanocarriers also possess the capabilities of protecting the drug payload or cargo from burst release, which would lead to the reduction of undesired off-target and side effects which can be caused by a burst release of drugs during circulation, leading to lesser nanoparticle usage [181,182,183]. Thus, rationalized designs for tailoring the specific applications of nanoparticles, optimization of nanoparticles’ pharmacokinetic features, and minimization of their off-target toxicological implications, have always been the critical criteria for translating these nanoparticles into clinics.

## 8. Current Clinical Status of Nanomedicine

Nanomedicine presents new health systems, paradigms, and chances, and several clinical products have already come into the market. Precise observational records and analysis or characterization have become quite crucial for all the scientific platforms and it becomes significant to not amplify the possible consequences for “nanotechnological” research and explorations; e.g., in its guiding written document the “US-FDA id not unconditionally judging the nanomaterial-based platforms or their applicability as such benignant or calumniatory.” The necessity for rich and string assessment has become quite evident, but cases for exceptional attention and care may not be that apparent [184,185]. The reliability or reproducibility of experimental data still presents a major and grave relevance for the entire safety establishment and clinic-based translational potentials of nanotechnology-derived medicines. These discourses around opportunities of possessing the checklist provide us the better chances in the context of the self-sufficient inspections and for reflection upon where all of us have been and what we have achieved in the recent past. While standardized approaches such as “one size would fit us all” may not present the best possible method, a personalized and specific checklist in terms of the newer breakthroughs or conceptions may quite well be conceived. At these stages and with quite limited and restricted patient results in nanobiotechnology and nanomedicine, promotion of the high-impact inventions for meeting immediate clinic-based requirements and addressing the central or fundamental translational problems is now becoming one of the topmost priorities [186,187].

In the recent past, explorations and developments in nanomedicine with regard to the interdisciplinary biological and nanotechnological arena have developed rapidly. The establishment of analysis and describing the standards can heighten the qualities as well as the wholeness of published explorations and research, promoting the reutilization and improving the outcomes, and can well modify the comparative assessments throughout various kinds of nanomedicines. Nano as a technology could well be seen as a solution to various problems in biomedical developments but has not been endorsed as the “performing nano for interest of the nano”. From these prospects, it could well be thought of as problem-derived nanomedicine or biomedicine conception, and has become a more significant requirement in bio and nano research for the improvement of success rates of nanomedicine translation into patient settings [188]. A successful and productive translation into patient settings has always been one of the primary and key objectives in nanomedicine explorations. For achieving these goals, researchers are required to make themselves able to bridge disruptions and lacunae among the preclinical and clinical formulation and developments. These processes can well be greased by describing the checklist for released data and reports which furnish and render the standard minimal information [185]. Several nanoformulations under current clinical stages have been briefed in Table 4 and the others that have been approved are placed in Table 5.

## 9. Conclusions and Future Perspective

There has been no doubt that nanomedicine therapy with continuously enhancing multifunctionalities will continue to exist in the coming times. As novel and more complicated nanoparticulate designs are appearing, appreciably improved tools, techniques, and methods for defining toxicity and biocompatibility will be required to be produced, specifically for those which could evaluate cellular, intracellular, and tissue and organ biocompatibility. While the contingents of matters concerning the scaleup of production and good manufacturing practices have often not been talked about, advanced and convoluted nanoparticles demonstrate that attempts towards surpassing good manufacturing and regulation constraints are under constant progress. Although several demanding situations have been existing for the translational potentials of multifunctional drug-delivery nanoparticles which have currently been employed for research and exploration purposes and as instruments for the accepted formulations for clinical medicines, their possible rewards should act as the driving force for their productive advancements and the continuous emergence of newer classes of nanoparticle-based therapies. The safety and toxicity assessment of medicinal nanoparticles need stimulation from several origins and subjects. The productive adaption and adjustments of the risk evaluation protocols to nanoparticles rely directly on the capability of individuals in industries, materials sciences, toxicology, and regulatory agencies understanding how their corresponding expertness accompanies the other aspects. There have been many clear acknowledgments of the values and significance of these multidisciplinary collaborative attempts for the improvements of nanoparticles’ toxicity assessment procedures. However, only limited approximations have been put into the practices yet, since regulatory authorities, the scientific community, and pharmaceutical industries perform from several distinct effronteries and have been quite engaged in their respective viewpoints.

In these newer times, evolving tools and techniques confront a more skeptical and postulating world—their advantages and gains to the societies need to be crystal clear, but also the scientific community including engineers needs to predict and qualify the probable risks related to their applications. In the case of nanobiomedicine and nanotechnologies, these hazards beleaguer the probable environmental entailments of widespread applications of customized and engendered multifunctional nanoparticles. Presently, nanoparticle exposure and health and safety concerns may not pose any considerable risks to public health and safety, since the most prevailing route of their exposure restricts the appraisal of their employability. However, with the increase in their quantities and kinds of customizations of drug-delivery nanoparticles applied in human populations, their potential regarding accidental environmental effects can further multiply correspondingly. Although it has become challenging to evaluate the hazards of engineered and customized nanoparticles before these may be well defined as commercial products, active research and explorations have become crucial for assuring a sustainable nanomedicine field. Overall, the applications of nanoparticles in biomedicine have the capability to create major effects on human safety. Therefore, it is advised to facilitate the advancements of personalized nanomedicine for particular patient groups, where nanotherapy can be well oriented by the patient’s specific genetic makeup as well as illness profiles. For example, illness-specific features and characteristics, viz. capillary permeabilities, expression levels of various cell surface receptors, and biomolecular pathways activity, can be assessed and applied for designing personalized medicine-based approaches. The physicochemical characteristics (viz. hydrodynamic diameter or nanoparticle size or their structures) of drug-delivery systems could further be tailored and customized in the context of the severity of illness for optimized remedial advantages. These concepts will considerably improve the ways in which patients are treated. However, for these phenomena to happen, there have still been several concerns which are required to be covered which include the basic comprehension of the pathophysiology of specific illnesses and nanobiological interaction of these multifunctional and customized nanoparticles in the patients, and several commercialization setbacks and roadblocks associated with manufacturing processes, costs, economics, and regulation criteria. Lastly, researcher communities are required to undertake the minimizations of the various complexities of nanoparticles and consider the ultimate dosage form for clinical applications, for the nanoformulation to possess the capability of translational potential into patient-based nanotherapeutic paradigms. Reduction and minimization of the complexities are needed for the pathophysiological or medicinal requirements and become paramount in the designing of nanoparticles and their synthetic schemes for the generation of clinically applicable nanosized therapeutic regimens.

The application of several analytical tools and methods which can use the complementarity, statistically strong, and significant data in the context of engineered nanoparticles characterization as well as biological localization or bioaccumulation is doubtlessly an assuring scheme to exploit these in both patients and environmental toxicity paradigms. Some of these tools and methodologies are employed for both environmental as well as patient-based samplings, whereas other techniques lie within specific domains of the nanobiomedical explorations. Clearly, as described above, a methodological cross-assessment and evaluation is quite obvious and needs to be immediately undertaken by researchers in nanomedicine disciplines. Beyond these obvious steps, a wider and fundamental intractability among the human’s nanomedicinal and environmental arenas must happen, since nanotoxicological data procured in these fields of explorations can be of considerable relevance to other fields as well. As the requirements for the development and advancements of specialized tools and techniques for the detection, characterization, and quantification of these customized and engineered nanoparticles in complicated systems can be shared by various fields, it has become imperative that these processes are channelized by collaborative attempts of scientific communities, by focusing on the analytic plannings and schemes adjustable for all arenas of nanoparticle and nanomedicine research.

## Figures and Tables

**Table 1:** Advanced nanoplatforms that are reported to be safe in different preclinical studies. In this table, we have covered the morphological characteristics of such nanoplatforms. The literature has reported a plethora of studies to demonstrate the safe and effective delivery of these nanocarriers; however, some of the recent examples have been briefed.

S. No	Nanoplatforms	Shape	Size	Coating Material	Ref.
1	Gold NPs	Spherical	~3 nm	Mercaptoethylsulfonate and heparin	[58]
2	Lipid NPs	Cone	60 nm	DLin-MC3-DMA ^1^	[59]
3	Mucus penetrating particles (MPPs)	Spherical	120–200 nm	Pluronics	[60]
4	LNPs-INT01	Spherical	<100 nm	LP01 ^2^	[61]
5	Silica NPs	Spherical/Fragments	~50 nm	Biodegradable disulfide	[62]
6	Biodegradable core-multishell NPs	Spherical	~20 nm	CMS ^3^	[63]
7	FA-CM NPs	Nanosheets	~130 nm	LDH ^4^ and MnO_2_^5^	[64]
8	MnO_2_ nanoplatform	Hollow spherical	170–180 nm	PEG	[65]
9	Chitosan NPs	Joint spherical	~200 nm	Lactobionic acid	[66]
10	Lipid-assisted NPs	Spherical	120–130 nm	Cationic Lipid (BHEM-Chol)	[67]
11	Polymeric NPs	Spherical	~50 nm	PLGA ^6^	[68]
12	Lipid-based NPs	Spherical	<12 nm	Meso-tritolyl-oxasmaragdyrin	[69]
13	Liposome Au-NPs	Spherical	~100 nm	DSPC and CHOL ^7^	[70]
14	Au-NPs	Rods; Cages; Spherical	100–150 nm	Nucleic acid; biodegradable polymer	[71]
15	Platinum-based NPs	Cages; Pyramid; Spherical	50–200 nm	biodegradable polymers	[72]

^1^ O-(Z,Z,Z,Z-heptatriaconta-6,9,26,29-tetraen-19-yl)-4-(N,N-dimethylamino)butanoate (DLin-MC3-DMA); ^2^ LP01: biodegradable ionizable lipid comprises of PEG-DMG; ^3^ CMS: hPG-PCL1.1K-mPEG2k-CMS; ^4^ CoAl: layered double hydroxides (LDHs) and manganese dioxide (MnO_2_); FA: folic acid; ^5^ MnO_2_: manganese dioxide; ^6^ PLGA: poly-(lactic-co-glycolic acid); ^7^ DSPC and CHOL: distearoyl phosphatidylcholine and cholesterol.

**Table 2:** Assessment of various safety and toxicity parameters with evaluation methods and their benefits.

S. No	Assessment	Mechanism	Methods	Advantages	Ref.
1.	Cytotoxicity	Cell proliferation/viability, morphological changes	xCELLigence	Observation of cell growth, cellular morphology, cell proliferation kinetics, and reproduction, with label-free techniques.	[86]
2.	ROS Levels	Depletion of antioxidant capacity	Fluorescent probes	Helps in the detection of H_2_O_2_ concentration with respect to epidermal growth factor (EGF) stimulation.	[87]
			Genetic approaches	It allows dynamic ROS monitoring by reversible oxidation.	[88]
			Nanoprobes	These have comparatively smaller sizes and could be injected via microinjection, lipofection, and TAT-protein delivery techniques.	[88]
			Nanoelectrodes	Super quick and highly sensitive as compared to traditional procedures.	[89]
3.	Genotoxicity	DNA breaks, altered bases, and chromosomal damage,	Automatic laboratory robot	Provide easy handling to 96 well plate to avoid DNA shear stress, precise dispensing of samples, and complete light protection.	[90,91]
			ToxTracker reporter assay	Identification of reactive oxygen species unfolded proteins as well as DNA damage.	[92]
4.	Immunotoxicity	Cytokine expression	ELISA, flow cytometry, and RT-PCR		[93]
			Human-based skin explant assays	Unique method.	[94]
5.	Carcinogenicity		Transgenic model	Carcinogenicity of carbon nanotubes and Ag NPs.	[95,96]
6.	Hepatotoxicity		3D microfluidics, 3D liver bioprinting, 3D organoid scaffolds	Minimize animal usage, cost-effective, and more similar to preclinical predictions.	[97]
7.	Omics-based toxicology		Genomics, transcriptomics, proteomics, metabolomics, lipidomics, and toxicogenomics	Highly sensitive for the detection of lower levels of nanoparticle exposure.	[98]

**Table 3:** Various factors for consideration for the translational development of nanomedicines (reproduced with permission from [81]).

Nano-Pharmaceutical Designs
**Key Considerations**
Route of administration Reduce complexity in formulation design Final dosage form for human use Biocompatibility and biodegradability Pharmaceutical stability (physical and chemical)
**Current Obstacles**
Large-scale production according to GMP standards ◦ E.g., Reproducibility, infrastructure, techniques, expertise and cost Quality control assays for characterization ◦ E.g., Size and polydispersity, morphology, charge, encapsulation, surface modifications, purity and stability
**Preclinical Evaluation**
**Key Considerations**
Need for validated and standardized assays for early detection of toxicity Evaluation in appropriate animal models of disease Adequate understanding of in vivo behavior, incl. cellular and molecular interactions ◦ Pharmacokinetics (absorption, distribution, metabolism and excretion) ◦ Pharmacodynamics (intracellular trafficking, functionality, toxicity and degradation)
**Current Obstacles**
Development of more specialized toxicology studies for nanomedicines Adequate understanding of the interaction of NNM with tissues and cells Adequate structural stability of NNM following in vivo administration Limited degree of accumulation of nanomedicines in target organs/tissues/cells
**Clinical Evaluation for Commercialization**
**Key Considerations**
Simplification of development pathways from invention to commercialization to minimize time and expense Evaluation of safety/toxicity in humans (acute and chronic) Evaluation of therapeutic efficacy in patients Optimal clinical trial design
**Current Obstacles**
Lack of clear regulatory guidelines specific for NNMs Complexity of NNM patents and IP Limited understanding of the biological interaction of NNM with the biological environment (incl. target site) in the body of patients

**Table 4:** Current clinical stages of various nanoformulations and nanomedicines (reprinted with permission from [81]).

S. No.	Name	Encapsulated Drug	Type of Formulation	Indication of Use	Clinical Status
1.	LiPlaCis	Cisplatin	Lipid-based nanomedicines	Advanced or refractory solid tumors, metastatic breast cancer and skin cancer	Phase I/II
2.	ThermoDox	Doxorubicin		Hepatocellular carcinoma, breast cancer	Phase I/IIIII
3.	SPI-077	Cisplatin		Ovarian cancer, relapsed/progressive osteosarcoma metastatic to the lung	Phase I/II/III
4.	Lipoxal	Oxaliplatin		Colorectal cancer, glioma	Phase II
5.	EndoTAG-1	Paclitaxel		Pancreatic cancer, 6.liver metastases, H7.ER2 and triple neg8.ative breast cance9.r	Phase II completed
6.	OSI-211	Lurtotecan		SCLC	Phase I/II completed
7.	LE-DT	Docetaxel		Solid tumors, pancreatic cancer	Phase I/II completed
8.	TKM-080301	siRNA against PLK1		Advanced hepatocellular carcinoma, solid tumors or lymphomas that are refractory to conventional therapies; colorectal, gastric, breast and ovarian cancers with hepatic metastases	Phase I/II completed
9.	Atu027	siRNA against PKN3		Advanced solid tumors, pancreatic cancer	Phase I/II completed
10.	2B3-101	Doxorubicin		Advanced solid tumors, brain metastases, lung and breast cancers, melanoma, malignant glioma	Phase I/II completed
11.	MTL-CEBPA	saRNA		Liver cancer	Phase I
12.	TLI	Topotecan		SCLC, ovarian cancer, solid tumors	Phase I
13.	MM-398 Onivyde	Irinotecan		Solid tumors, ER/PR positive and triple negative breast cancer, metastatic breast cancer with active brain metastasis, SCLC, metastatic pancreatic cancer	Phase I/II/III
14.	MM-302	Doxorubicin		Breast cancer	Phase I
15.	ATI-1123	Docetaxel		Advanced solid tumors	Phase I completed
16.	SGT-53	p53 pDNA		Solid tumors, recurrent glioblastoma	Phase I/II
17.	SGT-94	RB94 pDNA		Solid tumors, recurrent glioblastoma	Phase I, Phase II
18.	Anti-EGFR-IL-DOX	Doxorubicin		Solid tumors	Phase II
19.	RNL	Rhenium-186		Glioblastoma and astrocytoma (treatment and imaging)	Phase I/II
20.	Patisiran	siRNA		TTR-mediated amyloidosis	Phase I/II/III
21.	Paclical	Paclitaxel	Polymeric nanomedicines	Ovarian cancer	Phase III completed
22.	NK105	Paclitaxel		Gastric cancer	Phase III completed
23.	BIND-014	Docetaxel		NSCLC, solid tumors	Phase II completed
24.	CALAA-01	RRM2 siRNA		Solid tumors	Phase II terminated
25.	CRLX101	Camptothecin		NSCLC	Phase II completed

**Table 5:** Various nanoformulation-based medicines approved for the treatment of diseases and disorders (adapted from [189]).

S. No.	(Drug)-Formulation	Materials Employed	Advantages of Nanoformulation	Disease/Disorder	Approval Year
1.	Onivyde^®^ (Merrimack)	Liposomes containing the drug Irinotecan	Can increase the delivery to cancer localities; decrease systemic toxicities arising from side effects	Pancreatic Cancer	2015
2.	Adynovate (Baxalta)	Polymer and protein conjugation with (PEGylated-factor VIII)	For improving the stabilities of proteins by the PEGylation process	Hemophilia	2015
3.	Avinza^®^ (Pfizer)	Morphine sulfate	For increasing the encapsulation and bioavailability, to extend the drug release	Psychostimulant	2002 (2015)
4.	Invega^®^ Sustenna^®^ (Janssen Pharms)	Paliperidone palmitate	For the slower release of the injectables drugs with very low solubilities	Schizophrenia Schizoaffective Disorder	2009 2014
5.	Plegridy^®^ (Biogen)	Polymer conjugated with the proteins (PEGylated IFN-β 1a)	For improving the stabilities of the proteins by the process of PEGylation	Multiple Sclerosis	2014
6.	Ryanodex^®^ (Eagle Pharmaceuticals)	Dantrolene sodium	For fast administration at high doses	Malignant hypothermia	2014
7.	Cimzia^®^/certolizumab pegol (UCB)	PEGylated antibody (Certolizumab) fragments	For improving the blood residence time and higher stabilities in vivo	Crohn’s disease Rheumatoid arthritis Psoriatic Arthritis Ankylosing Spondylitis	2008 2009 2013 2013
8.	Abraxane^®^/ABI-007 (Celgene)	Anticancer drug paclitaxel bonded with albumin nanoparticles	For improving the solubility; better delivery to the cancers	Breast cancer NSCLC Pancreatic cancer	2005 2012 2013
9.	Marqibo^®^(Onco TCS)	Vincristine in the liposomal formulation	Improved delivery to cancer tissues; lesser systemic toxicities due to various off-target effects and side effects	Acute Lymphoblastic Leukemia	2012
10.	Krystexxa^®^/pegloticase (Horizon)	Protein and polymer conjugation (PEGylated porcine-like uricase)	For improving the stabilities of proteins by the PEGylation method; introducing the distinct mammalian proteins	Chronic gout	2010
11.	Mircera^®^/Methoxy PEG-epoetin-β	Chemical synthesis of the (erythropoiesis-stimulating agents)	For the improvement of the stabilities of the aptamers due to the process of PEGylation	Anemic conditions linked with the CKD	2007
12.	Macugen^®^/Pegaptanib	Antivascular endothelial growth factor aptamer in its PEGylated forms	For the improvements in the stabilities of aptamers due to the process of PEGylation	Age-related neovascular macular degeneration,	2004
13.	Somavert^®^/pegvisomant (Pfizer)	PEGylated human growth hormone-receptor antagonists in their PEGylated forms	For improving the stabilities of these proteinaceous components by the process of PEGylation	Acromegaly	2003
14.	Eligard^®^ (Tolmar)	Leuprolide acetate forms and their polymers (viz. (poly-DL Lactide-coglycolides))	For the aim of controllable drug delivering of therapeutic payloads and imparting the long blood residence times	Prostate Cancer	2002
15.	Neulasta^®^/pegfilgrastim (Amgen)	granulocyte colony-stimulating factor proteins in tier PEGylated forms	For improving the stabilities of the proteinaceous components by the PEGylation process	Cancer chemotherapy-induced neutropenia	2002
16.	Pegasys^®^ (Genentech)	Interferon-α 2a proteins in their PEGylated forms	For imparting the enhanced stabilities to these proteinaceous components by the process of PEGylation	Hepatitis B; Hepatitis C	2002
17.	PegIntron^®^ (Merck)	Interferon-α 2b proteins in their PEGylated forms	For improving the stabilities of proteinaceous parts by the process of PEGylation	Hepatitis C	2001
18.	Renagel^®^ [sevelamer hydrochloride]/Renagel^®^ [sevelamer carbonate] (Sanofi)	Poly-(allylamine-HCL)	Improved blood residence times and effective drug delivery	Chronic kidney disease	2000
19.	Copaxone^®^/Glatopa (Teva)	Randomized copolymers of L-glutamine, L-alanine, L-tyrosine, and L-lysine	Larger amino acid-based polymers possessing regulated molecular weights as well as body clearance features	Multiple Sclerosis (MS)	1996
20.	Adagen^®^/pegademase bovine (Sigma-Tau Pharmaceuticals)	Adenosine-deaminase enzymes in their PEGylated forms	For the improvements in the blood-circulation times and reduced immunogenic outcomes	Severe combined-immunodeficiency disease	1990
